# Neonatology Specialty Grand Challenge

**DOI:** 10.3389/fped.2013.00013

**Published:** 2013-07-08

**Authors:** John Steven Torday

**Affiliations:** ^1^University of California Los AngelesLos Angeles, CA, USA

Historically, though high infant mortality rates were recognized by the British medical community at least as early as the 1860s, the advent of neonatal intensive care is relatively recent. In 1898, Dr. Joseph B. De Lee (1) established the first premature infant incubator station at the Chicago, Illinois Lying-in Hospital. The first American textbook on prematurity was published in 1922 (2). It was not until 1965 that the first American newborn intensive care unit (NICU) was opened at Yale University, but more importantly, only a few years later Egon Diczfalusy conceptualized the fetoplacental unit (3), which established that the fetus actively participates in its own physiologic development *in utero*. This represented a fundamental break with Victorian attitudes toward maternal confinement during pregnancy, and set the bar for reproductive physiology to this day.

Such breakthroughs as Helen Taussig's surgical approach to the treatment of the Tetralogy of Fallot (4); the Liley Score for hyperbilirubinemia (5); the L/S ratio (6), Continuous Positive Airway Pressure ventilation (7), antenatal steroids (8), and postnatal exogenous surfactant to prevent and treat Respiratory Distress Syndrome (RDS) (9) all led inexorably to the fetus as a patient. It was the advent of antenatal steroids for lung immaturity that was the call to action for the Neonatology Community – the realization that there was a way to proactively affect the outcome of preterm birth, not merely passive watchful waiting (For example, Dr. Gluck's dictum that if the L/S ratio were less than 2:1, to wait for 2 weeks and retest). That challenge set the bar, and it remains to this day, given all of the ancillary effects of antenatal steroids on multiple organs and systems.

The origins of Neonatology as a formal discipline were sown by investigators passionately curious about the limits of human physiology. Clement Smith would relate his story of how he and his pediatric colleagues were allowed to round on preterm infants at the Boston Lying-In Hospital in the 1950s, initially observing, and then providing minimal nourishment (a little glucose on the tongue) and comfort (warming lamps). One of Professor Smith's more ambitious, forward-thinking, and observant Fellows, ME Avery, would express to her students how dissatisfied she was with the diagnosis of Hyaline Membrane Disease (HMD) as an inevitable post-mortem consequence of pulmonary obstructive disease. Instead, she took the initiative of studying lung surfactant in these infants, hypothesizing that they were surfactant deficient. She and Jere Mead demonstrated that the lung washings of preterm infants who died of HMD were deficient in surface tension-reducing activity in their airways (10), subsequently championing one of the first U.S. clinical trials for the use of antenatal glucocorticoids to stimulate lung surfactant production, following on the first clinical use of antenatal glucocorticoids by Liggins and Howie in New Zealand (11). As a result of this intervention, the mortality rate due to RDS among infants less than 32 weeks gestation fell dramatically, but the morbidities of preterm birth – bronchopulmonary dysplasia, necrotizing enterocolitis, retinopathy of prematurity, intraventricular hemorrhage, cerebral palsy – necessarily increased as a direct result of the increased survival, leaving the newly established discipline of Neonatology with the tandem challenges of further reducing morbidity, while deciphering the underlying principles of physiology to predict and prevent the sequelae of preterm birth.

There is a long history of systematic approaches to determining where along the normal course of development any given preterm infant is at the time of birth. In 1952 Dr. Virginia Apgar described the Apgar scoring system (12) as a formalized means of evaluating a newborn's physiologic condition at the time of birth. And then there were Lula Lubchenko's standardized birthweights (13), which provided objective data for calibrating gestational age at birth. More recently, Doug Richardson (14) had devised the SNAP score. All of these efforts are designed to try and circumscribe the emergent and contingent character of preterm birth, much like the description of the process of Evolution itself, which has been equally elusive for the very same reasons. Identifying the predictors of neonatal physiology and well-being remains a challenge.

The only advance in Neonatology based on *a priori* scientific principles of fetal physiology and adaptation was the discovery by Avery and Mead (10) that RDS was due to lung surfactant deficiency at birth. All of the other treatments and procedures were derived *a posteriori* from standard pediatric physiology reduced to its application to the preterm newborn. Over time, the discipline became aware that preterm infants are not merely small term infants, they are fetuses who have not achieved their full physiologic capacity. We have yet to discover the underlying cause of preterm birth, or the fundamental phylogenetic mechanisms of adaptive physiology. We have not made any progress in ascertaining *the first principles of human physiology* as the basic scientific underpinning Neonatology, other than to amass volumes of clinical information as an empiric guide for managing preterm infants – it must be underscored that there is a fundamental difference between information and knowledge.

Now, armed with molecular biologic methods – genomics, proteomics, metabolomics, phenomics, etc. – we must determine what principles determine the processes of physiologic adaptation at the cell-molecular, tissue, organ, systemic, and organismal levels if we are to fulfill the ultimate, and lofty goal of Neonatology – for every newborn to achieve its genetic destiny. *That is the Grand Challenge*. When Mary Ellen Avery invited me as a basic scientist to join her research group as it transitioned from McGill University to Harvard Medical School in 1974, she said to me “we need to provide science as the foundation for Neonatology, or we could do a lot of harm in the name of good.” Frontiers in Pediatrics: Neonatology is dedicated to the on-going reporting and encouragement of that effort.
